# Correction: Better start to bilingual development: Bridging parental beliefs and science through early intervention for Polish families living in Norway

**DOI:** 10.1371/journal.pone.0351369

**Published:** 2026-06-09

**Authors:** The PLOS One Staff

The ORCID iDs are missing for the fifth, sixth and eighth author. Please see the authors’ respective ORCID iDs here:

- Author Magdalena Krysztofiak’s ORCID iD is: 0000-0002-3160-9148 (https://orcid.org/0000-0002-3160-9148).
- Author Magdalena Łuniewska’s ORCID iD is: 0000-0001-5504-9766 (https://orcid.org/0000-0001-5504-9766).
- Author Nina Gram Garmann’s ORCID iD is: 0000-0002-2932-7547 (https://orcid.org/0000-0002-2932-7547).

In the Author Contributions, Magdalena Łuniewska should be listed as one of the persons who contributed to: Funding acquisition, Investigation and Writing – original draft.

The publisher apologizes for the errors.
