# Supplementary material for: Better start to bilingual development: Bridging parental beliefs and science through early intervention for Polish families living in Norway
Source: PLoS One. 2026 Apr 9;21(4):e0346160. doi: 10.1371/journal.pone.0346160 (PMC13065072; doi:10.1371/journal.pone.0346160)
Supplement: Attachment — Submitted filename: Response to Reviewers.pdf [file pone.0346160.s003.pdf]

Response to Reviewers

Manuscript “Better start to bilingual development: Bridging parental beliefs and science through early intervention for Polish families living in Norway”

Editor Comments

Comment 1

How can both an IV and a DV use the same measure? It could be pre- post for that third variable but at entry isn’t it the same?

Comment 6

The regression needs to be more detailed in how you conducted entering the predictor variables. The best one and would add to your paper is hierarchical where you add the ones that have the highest prediction first and then one by one after that. The benefit of this is that you can get R square change scores. In that way you can tell how much variance is explained by the intervention itself and then how much by having other children etc. It provides much more information.

Response

To Comment 1: In the previous version of the paper, the regression analyses examined the gain from participating in the intervention as the dependent variable. This gain score was calculated by subtracting the pretest score (TP1) from the posttest score (TP2): gain = TP2 − TP1. The pretest score (TP1) was also included as one of the independent variables. Although the gain score is mathematically related to the pretest score, the posttest score (TP2) is also conceptually connected to it, albeit not in a purely mathematical way.

Nevertheless, we agree that replacing the dependent variable with the raw posttest score (TP2) results in a clearer model that better aligns with standard and statistically appropriate practices for this type of study. For this reason, we decided to add the analysis that examines the *score at posttest* as a dependent variable. In the manuscript, we refer to this analysis as ***non-preregistered regression analysis 1***. This decision also required corresponding adjustments to other parts of the paper (e.g. the research question, Discussion and Conclusions).

Since the analysis of the predictors of the gain from participating in the intervention provides different information and was preregistered, we retained it in the paper. In the current version of the manuscript, we refer to this analysis as ***preregistered regression analysis 2***.

Regarding **Comment 6**, in the previous version of the paper the predictors were entered into the model simultaneously. In the current version, we followed the recommendation to add the predictors step by step.

Please note that in the analysis of gains from the intervention (preregistered regression analysis 2), we included only data from participants who received the experimental intervention. Because we were interested in identifying factors that influenced gains resulting specifically from the experimental condition, only participants assigned to that condition could be examined. Consequently, the group factor was not included in this model.

In contrast, the analysis now added to the paper (non-preregistered regression analysis 1) examines the factors influencing the level of alignment between parental beliefs and science at posttest. This allowed us to include data from all participants and to treat the group factor as an independent variable, thereby examining the proportion of variance explained by participation in the experimental versus control condition, as advised in your comment.

One final change we made was to replace the variable "number of children in the family" (previously treated as a numeric predictor) with "prior parenting experience (raising an older child: yes/no)." We observed that only seven participants (8.3% of the total sample) were raising more than one child, which made the numeric treatment of this variable inappropriate. A dichotomous coding therefore provided a better fit for the data. This modification resulted in slight changes to the conclusions drawn from the analysis of the predictors of gains from the intervention (preregistered regression analysis 2).

All changes to the regression analysis, as well as the corresponding modifications in other parts of the manuscript, are described below.

Changes in the manuscript

The changes described above resulted in a number of changes in the following sections:

Abstract (lines: 37-41, 44-45)

Introduction. The current study subsection (lines: 225, 234-241, 256-261)

Methods. Participants (lines: 355-360)

Methods. Variables and measures (lines: 463-465)

Methods. Analytic strategy (lines: 488-490, 503-542, 532-533)

Results. Predictors of parental beliefs' alignment with science following participation in the experimental or control intervention: non-preregistered regression analysis 1 (New subsection, lines: 599-650)

Results. Predictors of the increase in parental beliefs alignment with science: preregistered regression analysis 2 (lines:653-660, 674-695)

Discussion (lines: 792-856, 863-865, 867-868, 870-871, 876-878)

Comment 2

Table 2 descriptive statistics on what variable?

Response

The statistics in Table 2 refer to the results of the questionnaire on beliefs about language development and bilingualism. The questionnaire is used to measure the extent to which the participant's beliefs are consistent with scientific evidence. We have changed the description of the table and the reference to the table in the text to make this clearer.

Change in the manuscript

Lines 565-566:

- Original: see Table 2 for descriptive statistics for both groups at both time points
- Revised: see Table 2 for descriptive statistics of parental beliefs about language development and bilingualism across groups and timepoints.

Lines 584-585:

- Original: Table 2. Descriptive statistics for participants' performance at pretest and posttest in experimental and control group.
- Revised: *Descriptive statistics for questionnaire scores on parental beliefs about language development and bilingualism across groups and timepoints (TP1, TP2)*

Comment 3

I think you mean a Bonferroni post-hoc (which controls for increasing alpha) and not a Bonferroni correction

Response

We agree that "Bonferroni post-hoc test" is a more precise term for the analysis we conducted than "Bonferroni correction".

Change in the manuscript

We have made changes in five instances in the Results section (lines: 567, 573, 580, 592, 722, 729, 734, 739, 762), for example:

- Original: Post hoc tests using Bonferroni correction revealed that the groups did not differ significantly (…).
- Revised: Bonferroni post-hoc tests revealed that the groups did not differ significantly (…).

Comment 4

effect increased with time but

There were no significant differences between the posttest and the retention test. For the control group, no significant differences were observed between any time points

effect was seen in the control group but

For the control group, no significant differences were observed between any time points

These two in red from the discussion do not seem to agree with your results

Then

As the effect is found in both the experimental and the control group, general reasons, like being exposed to scientifically based knowledge after giving birth or becoming interested in scientifically based knowledge after attending workshops, cannot be ruled out as explanations.

I don't see the effect in the control group

Response

Thank you for pointing out the mistake in the Discussion section. There were no significant differences over time in the control group, however, the Discussion incorrectly included information and interpretations suggesting otherwise.

Change in the manuscript

We removed the incorrect part of the Discussion (see the removed paragraph below). The first sentence was slightly rephrased and moved to lines 779-783.

Original [now deleted completely]: In contrast to the results of previous research [65], the effect of the language intervention was maintained over time and even increased with time (in the experimental group). This increase was seen not only in the experimental, but also in the control group (although the effect was significantly larger in the experimental group). This might be a result of repeated testing with the same tool (the same questions in a different order), or being exposed to evidence-based knowledge after the child's birth (independently of our workshop), or searching for evidence-based knowledge either as a consequence of the workshops or as a consequence of participants' characteristics, e.g. having relatively high-level education. Observing one's own child for 9 months may also have provided participants with additional knowledge. This is confirmed by the fact that in the pre-test, those who had older children scored higher than those expecting their first child. As the effect is found in both the experimental and the control group, general reasons, like being exposed to scientifically based knowledge after giving birth or becoming interested in scientifically based knowledge after attending workshops, cannot be ruled out as explanations. This also shows the methodological importance of including a control group (preferably both an active and a passive one) when carrying out interventions. Had we also included a passive control group in the design, not receiving any intervention at all, we might have gained further insights into the underlying reasons behind the increase in both groups.

Comment 5

Data—please provide the ANOVA tables that match the two analyses that you completed

Response

We have added four ANOVA tables (one with the ANOVA main effects statistics and one with Bonferroni post hoc test for each of the ANOVA models).

Change in the manuscript

Lines 587-592:

Table 3

Preregistered mixed ANOVA Analysis 1: Results on intervention effectiveness

**Effect**	**F**	**df1**	**df2**	***p***	**η2**
**Group**	13.56	1	74	< .001	.13
**Time point**	31.79	1	74	< .001	.08
**Group x Time point**	11.77	1	74	< .001	.03

Table 4

Bonferroni post-hoc test results on intervention effectiveness

**Group comparison**	**Mean difference**	***p***
TP1: Experimental vs control	3.2	ns
TP2: Experimental vs control	9.4	< 0.001
Experimental: TP1 vs TP2	7.9	< 0.001

Control: TP1 vs TP2	1.9	ns

Lines 756-761 :

Table 9

Non-preregistered mixed ANOVA analysis 2: results assessing the maintenance of intervention effects

**Effect**	**F**	**df1**	**df2**	**p**	**η2**
**Group**	7.28	1	40	.010	.12
**Time point**	16.39	2	80	< .001	.09
**Group x Time point**	1.86	2	80	.163	.01

Table 10

Bonferroni post-hoc test results for the maintenance of intervention effects

**Group comparison**	**Mean difference**	***p***
TP1: Experimental vs control	3.6	ns
TP2: Experimental vs control	7.6	0.009
TP3: Experimental vs control	6.9	0.04
Experimental: TP1 vs TP2	6.9	0.041

Experimental: TP2 vs TP3	0.7	ns
Experimental: TP1 vs TP3	7.6	0.022
Control: TP1 vs TP2	2.9	ns
Control: TP2 vs TP3	1.5	ns
Control: TP1 vs TP3	4.4	ns

Reviewers' Comments

Comment 7

Reviewer #1: The authors have adequately addressed the concerns from previous editors. I recommend they review the article one last time for English cohesion and grammar.

Response

Thank you for your positive feedback and suggestion regarding language. The whole manuscript was revised with regard to the English language: wording, cohesion, and grammar.

Change in the manuscript

The text was proofread and refined for grammar and style by human agents. We also used AI tools (ChatGPT and DeepL) to further support proofreading in same parts of the text. All generated suggestions were manually reviewed, and we take full responsibility for the final version of the manuscript. Editorial changes are not highlighted in the current revised version to ensure that the substantive revisions, marked in blue, remain clear to the Editor and Reviewers.
