# Supplementary material for: Better start to bilingual development: Bridging parental beliefs and science through early intervention for Polish families living in Norway
Source: PLoS One. 2026 Apr 9;21(4):e0346160. doi: 10.1371/journal.pone.0346160 (PMC13065072; doi:10.1371/journal.pone.0346160)
Supplement: Attachment — Submitted filename: Response to Editor.pdf [file pone.0346160.s005.pdf]

Response to Reviewers

Manuscript "Better start to bilingual development: Bridging parental beliefs and science through early intervention for Polish families living in Norway"

Thank you for all your comments and ideas on how to further improve our manuscript, which we very much appreciate. We provide our answers in green, after each comment or after a group of comments (if connected). When we cite parts of our manuscript modified according to your suggestions, the changes made directly in the text are provided in blue. Please also note that if we delete some parts according to your suggestions, we acknowledge it here (in the response to Reviewers), but it is no longer visible in the modified version of the manuscript.

Editor Comments

Comment 1

The paper is much improved but includes sections and statements that do not provide new knowledge. The Introduction section of this manuscript is one of those sections. I suggest removing it.

Comment 2

The statement before is on lines 84-86. It basically repeats the sentence above it. Delete.

Comment 3

This paragraph is basically redundant to other sections:

Most of the research on parental interventions available in the literature is addressed 138 to monolingual populations. Some studies describe language-related interventions aimed at 139 bilingual families of low SES [e.g. Latino communities in the US, 38], or where multilingual 140 families constituted a significant proportion of participants [e.g. 39–42]. However, the studies 141 on bilingual families focused solely on the majority language of the children. Hence, 142 language interventions aimed at preventing language difficulties have yet to focus on families 143 planning to raise children in bilingual environments [4

Comment 4

This sentence is also redundant:

This is consistent with the studies showing that parental 177 knowledge about language and cognitive development at the time of their child's birth 178 influences caregiving behaviours several months later [17]

Comment 5

This sentence is also redundant:

This is consistent with the studies showing that parental 177 knowledge about language and cognitive development at the time of their child's birth 178 influences caregiving behaviours several months later [17]

Comment 6

This one is redundant of the sentence before it:

Dialogic reading has been implemented as 194 a standalone approach in numerous parent-oriented language interventions.

—delete

Comment 7

197 to 201 is again redundant –please delete

Comment 8

Delete redundant:

Our intervention was developed based on the research on communication 210 and caregiving behaviours supporting language development

Response

Thank you for pointing out all the redundancies.

Changes in the manuscript

The redundant paragraphs or sentences were removed according to your advice.

Comment 9

Line 128 - add 'and' before Comment

Response

Added.

Comment 10

Evidence-based recommendations for parents and parental interventions—this heading is not needed---the section is extremely short and can be one paragraph and integrated into the section before this heading

Comment 11

Again, the heading is not needed nor is the above paragraph (138 to 143)

Response & changes in the manuscript

Headings removed.

Comment 12

Then the next paragraph (referring to: *Up to date, there are few interventions…*) makes your case but you stated that a few studies and you do not cite them. Please add citations to that statement.

Response

We added the citation for the systematic review (Larson et al., 2020) addressing this issue along with two newer publications directly addressing their interventions to bilingual populations.

Changes in the manuscript

Line 127 - 130:

Up to date, there are few interventions tailored specifically for bilingual families, providing support that directly addresses the challenges associated with raising children in a multilingual environment and supporting their home language development [see 38 for the review; see also 28,29].

Comment 13

Some parts do not have citations (i.e., first sentence)

Response

Text was reviewed once again. The missing citations were added.

Changes in the manuscript

Lines 127-130:

Up to date, there are few interventions tailored specifically for bilingual families, providing support that directly addresses the challenges associated with raising children in a multilingual environment and supporting their home language development [see 38 for the review; see also 28,29].

Lines 781-783:

In contrast, for participants with lower levels of education, active engagement may play a more critical role [57]

Comment 14

163 to 170 discusses a topic you hsd earlier---please move it to that area

Referring to:

Some studies directly measured the changes in parental knowledge or beliefs due to taking part in interventions. All of them (internet version of PALS [46]; Thirty Million Words-Well Baby intervention [47]; 3Ts – Tune In, Talk More, Take Turns – Home Visiting [48,49]) showed that taking part in the interventions resulted in the increase of parenting knowledge [46] or knowledge about early childhood cognitive and language development [47–49]. In most cases ([48,49] and in high intensity training described by List and collaborators [47]), the increase in knowledge was also related to enriched parent-child interactions.

Response

This section focuses on measuring parental knowledge in any parental interventions having a language component. The previous part discussed in detail all outcomes of the interventions directly addressing the bilingual context. Only one of them overlap (Feil et al., 2020). We agree that this section should appear earlier in the text, so we have moved it before the paragraph describing interventions for bilingual populations in detail. We also included some minor changes to the paragraph describing study by Feil et al. (2020) not to repeat the same information.

Changes in the manuscript

Lines 103-111:

There is a substantial body of research evaluating parental interventions that include a language component [see 27 for meta-analysis]. Some of them directly measured the changes in parental knowledge or beliefs due to taking part in interventions. All of these studies (internet version of PALS [28]; Thirty Million Words-Well Baby intervention [29]; 3Ts – Tune In, Talk More, Take Turns – Home Visiting [15,30]) showed that taking part in the interventions resulted in the increase of parenting knowledge [28] or knowledge about early childhood cognitive and language development [15,29,30]. In most cases ([15,30] and in high intensity training described by List and collaborators [29]), the increase in knowledge was also related to enriched parent-child interactions.

Lines 142-146:

The study included a control group and showed that mothers who participated in PALS not only demonstrated a significantly greater increase in parenting knowledge but also improved language-supportive behaviors. Children's language outcomes were also improved compared to those in the control group, who received attention-control training.

Comment 15

Not a paragraph:

We hypothesised that the alignment of parental beliefs with scientific evidence on 232 language development would increase significantly in the group that received the language 233 intervention compared to the control group.

If it adds new information, integrate it into the paragraph below

Response & changes in the manuscript

We have merged the sentence with the subsequent paragraph, as suggested. Because this was the first place in the manuscript where our hypothesis was explicitly stated, we decided to retain it in the text to ensure clarity for the reader.

Comment 16

Line 267 birthing (how it is discussed in English)

Response

We have revised the sentence to improve clarity and avoid any potential ambiguity.

Changes in the manuscript

Lines 234-236:

We targeted parents born in Poland who were living in Norway and planning to remain there long-term. Eligible participants were either expectant parents or parents of infants aged ≤ 3 months.

Comment 17

On 338 you said of the 95—84 attended at least one session in person

Then in the next paragraph you state—of the 84—55 attended three synchronously

Please make this into a table as it is difficult to follow as written

Response

Thank you for this helpful suggestion. We realise that the flow of participant numbers across attendance categories may have been difficult to follow in the text. To improve clarity, we have now summarised session attendance in Table 1. and simplified the description in the manuscript. We hope that right now it is clear that 84 refers to participants who completed both pretest and posttest measures (out of 95 who attended at least one session).

Changes in the manuscript

Lines 307-316:

Out of the 95 participants who attended at least one of the three intervention sessions synchronously (session attendance is summarised in Table 1), 84 completed both the pretest and posttest measures and were therefore included in the current analyses. The final

randomised sample consisted of $N = 76$ participants ($n = 40$ in the experimental group and $n = 36$ in the control group). An additional $N = 8$ non-randomised participants were included only in the exploratory analyses (thus $N = 84$ in total). All participants who did not attend all intervention sessions were given the opportunity to review the material independently using PDF handouts.

Table 1 *Intervention session attendance by study group (N = 84)*

Number of sessions attended synchronously	Randomised		Non-randomised – language workshop	Total
	Experimental – language workshop	Control – sleep workshop		
Three (all)	24	24	7	55
Two (one missed)	11	8	0	19
One (Two missed)	5	4	1	10

Comment 18

Line 460 (Cronbach's alpha = .83) please add the)

Response & changes in the manuscript

Added

Comment 19

Regressions do not have IV and DVs. They had predictors and outcomes—please rephrase

Response

Thank you for this comment. We have revised the terminology throughout the manuscript and now refer to predictors and outcome variables in the context of the regression analyses.

Changes in the manuscript

Line 429:

In the regression analyses, five predictors were taken into account: (...)

Lines 437-439:

The second of those variables, parental beliefs at the study entry, was assessed using the same questionnaire as the outcome variable [50].

Lines 449-450:

The last two predictors (education and prior parenting experience) were collected in the demographic questionnaire.

Lines 471-473:

The outcome variable was the score on the beliefs questionnaire [50] in the posttest (TP2). The predictors were: (...)

Lines 492-495:

The outcome variable was the increase in the score on the beliefs questionnaire [60] between pretest (TP1) and posttest (TP2). This increase was calculated as the difference of scores between TP2 and TP1 scores. The predictors were: (...)

Lines 569-570:

The outcome variable was the beliefs questionnaire score at posttest (TP2 score).

Lines 620-621:

The outcome variable was the increase in score from TP1 to TP2 (the gain).

Comment 20

you have some squares in the stats (line 547) but when I copy it is comes out as β-squared---please check

Response

Thank you for noting this issue. The statistic refers to the chi-square (χ^2) test. The symbol appears to have been altered during file conversion, which led to the formatting error. We have verified that in the currently uploaded DOCX files the statistic is displayed correctly.

During the PDF-building process in Editorial Manager the problem appears again; however, this stage is beyond our control.

Comment 21

674---given that only the first model added do not include the other models as they are not contributing to the effect

Response

Thank you for bringing this to our attention. We have removed from the text the details of the models that did not significantly improve model fit. We made a similar revision in the description of the non-preregistered regression analysis 1, removing the details of the model that did not significantly contribute to the explained variance.

Changes in the manuscript

Line 639-642:

In Step 1, the model including the pretest score was significant and explained 51.3% of the variance in the increase in score ($R^2 = .51$, adj. $R^2 = .50$), $F(1,46) = 48.45$, $p < .001$. The addition of engagement (Step 2), education (Step 3), and prior parenting experience (Step 4) did not improve model fit (see Table 8 for summaries of each model).

Line 581-582:

Engagement introduced in Step 3 did not significantly increase the explained variance (see Table 6 for model summary).

Comment 22

No data in the discussion---the discussion is not a repeat of the results so interpret the importance of the findings in relationship to the lit review and any other possible new discoveries

Response

Thank you for this helpful comment. In the revised version, we have removed the remaining descriptive references to statistical models from the Discussion section to avoid repetition of

the Results. We have also added several interpretative passages that clarify their theoretical implications.

Changes in the manuscript

Line 765-776:

The fact that participation in the experimental workshop contributed more to having beliefs aligned with science at the end of study, than participation in the control intervention, confirmed the effectiveness of the intervention. Baseline alignment with science score significantly predicted post-intervention beliefs as well, indicating that participants' initial beliefs remained a strong determinant of subsequent scores. This finding is consistent with prior evidence demonstrating substantial temporal stability in knowledge-related constructs [55]. Although individual engagement did not account for additional variance, its predictive role became evident once demographic factors—particularly education—were taken into account. This pattern suggests that engagement and education share variance, and that controlling for educational background clarifies the unique contribution of engagement to post-intervention outcomes. The effect of engagement may be obscured by educational attainment.

Line 791-797:

This effect is difficult to interpret. On the one hand, it is possible that participation in the intervention helped first-time parents align their beliefs with scientific evidence to a level comparable to that of more experienced parents. An alternative explanation is that prior parenting experience was moderately correlated with pre-existing beliefs (i.e., the score at TP1). Therefore, the effect of prior parenting experience may already have been accounted for when the TP1 score was introduced in the second step of the model.

Line 804-812:

The lack of predictive power of individual engagement for gains from the intervention suggests that its significance in explaining the change in parental beliefs may be tied to pre-existing knowledge, rather than to the anticipated causal mechanism whereby greater engagement would translate into greater learning. However it should be noted that this analysis was conducted on a smaller sample size compared to non-preregistered regression analysis 1, as it was restricted to participants in the experimental condition and did not include data from the control group. Consequently, the reduced sample size may have limited statistical power, potentially obscuring effects that would have been detectable in a larger sample.

Line 830-841:

Contrary to our predictions, prior parenting experience did not predict either participants' post-intervention beliefs or their gains from the intervention. The reason for this might be that parents expecting their first child presented a slightly but significantly lower level of alignment with scientific evidence at the pretest compared to those who already had children. This may reflect fewer opportunities and less motivation to observe young children, ask questions about their development, and search for evidence-based guidelines. As a result, any advantage associated with parenting experience may already have been captured by participants' initial knowledge, entered in earlier steps of the model. Consistent with previous research [58], parenting knowledge varies with experiential and sociodemographic factors, with more experienced parents generally demonstrating higher domain-specific understanding. In this sense, prior experience did not exert an independent effect beyond baseline knowledge.

Comment 23

You have multicollinearity in your data—in 868

From AI

a statistical phenomenon in regression analysis where two or more independent variables are highly correlated, meaning one can predict another, creating redundant information that makes it difficult to determine each variable's individual effect on the outcome variable

it is like parental income and free lunch in the US. To get free lunch – parents have to have low income—so they are the same variable

Response

Thank you for raising this important point. As indicated in the supplementary analyses, TP1 scores were moderately correlated with both education and prior parenting experience, suggesting partial shared variance among predictors. In the revised Discussion, we have not only clarified that the non-significant effects of some predictors may reflect overlapping informational resources rather than the absence of substantive influence, but also added a brief note directly acknowledging that multicollinearity may have attenuated some individual regression coefficients.

Changes in the manuscript

Line 842-850:

It should be noted that some degree of overlap between predictors was confirmed by supplementary correlation analyses. They indicated that TP1 scores were positively correlated with both education and prior parenting experience. This pattern suggests partial shared variance among these variables, which may have reduced the apparent unique contribution of education or prior experience in multivariable models. Thus, the non-significant effects of some predictors should be interpreted cautiously, as they may reflect overlapping informational resources rather than the absence of substantive influence. Although the correlations were moderate rather than high, they indicate that multicollinearity may have attenuated some individual regression coefficients.
