# Supplementary material for: Better start to bilingual development: Bridging parental beliefs and science through early intervention for Polish families living in Norway
Source: PLoS One. 2026 Apr 9;21(4):e0346160. doi: 10.1371/journal.pone.0346160 (PMC13065072; doi:10.1371/journal.pone.0346160)
Supplement: Attachment — Submitted filename: Response_to_Editor_auresp_4.pdf [file pone.0346160.s006.pdf]

**Response to Reviewers**

Manuscript "Better start to bilingual development: Bridging parental beliefs and science through early intervention for Polish families living in Norway"

Thank you very much for your careful reading and for pointing out the mistakes that were missed during our work on the revised version. We have corrected all issues you indicated and hope that no further errors remain.

## Editor Comments

### Comment 1

1--Line 151 page 5 & needs to be replaced with the word 'and'

***Response***

Replaced.

***Changes in the manuscript***

Line 118-122:

Research on the relation between certain parental behaviours and children's language development has resulted in recommendations for caregivers [32] and strategies that can be implemented to promote language development in children, e.g. *Support, Ask, Expand* by Boyce et al. [33], *1,2,3, Tell Me What You See*, by Brannon and Dauksas [34], and *Comment, Ask and Respond* by Cole et al. [35].

### Comment 2

2--line 180 page 8 there are two periods. .. please delete one

***Response***

Deleted

***Changes in the manuscript***

Line 179-180:

However it does not meet the complex needs of bi- and multilingual families.

**Comment 3**

3--line 37 page 16 since refers to time and you have used that in a sentence that the semantic meaning is cause. Can you replace that with either 'as' or 'because'. (honestly I would not flag this without the others)

***Response***

Replaced.

***Changes in the manuscript***

Line 357-358:

All parents also filled in a questionnaire on children’s sleep patterns, as this was the theme for the control group intervention.
