# Supplementary material for: Better start to bilingual development: Bridging parental beliefs and science through early intervention for Polish families living in Norway
Source: PLoS One. 2026 Apr 9;21(4):e0346160. doi: 10.1371/journal.pone.0346160 (PMC13065072; doi:10.1371/journal.pone.0346160)
Supplement: Attachment — Submitted filename: Response_Reviewer_Better Start.docx [file pone.0346160.s001.docx]

**Response to the Reviewer**

Manuscript “Better Start to Bilingual Development: Bridging Parental Beliefs and Science Through Early Intervention for Polish families living in Norway”

[The changes in the text are highlighted in blue]

## **Reviewer 2.**

## Comment 1

**Comment of the Reviewer:**

The theoretical background is well laid out in the introduction. There are places, though, where it is difficult to distinguish between types of interventions referred to (behavioral interventions or belief interventions).

**Response:**

Thank you for pointing out this limitation. We acknowledge that the section lacked clear information about the interventions that measured parental beliefs or knowledge. We added the paragraph that explains that. We also added the more detailed information in the paragraph describing the studies on one of the interventions (PALS).

**Change in the manuscript:**

We added the following paragraph in the subsection *Previous research on parental interventions* (lines 156-163)*:*

Some studies measured directly parental knowledge or beliefs changes due to taking part in interventions. All of them (internet version of PALS [[46]](https://www.zotero.org/google-docs/?pBUC8M); Thirty Million Words-Well Baby intervention [[47]](https://www.zotero.org/google-docs/?tp84N9); 3Ts – Tune In, Talk More, Take Turns – Home Visiting [[48,49]](https://www.zotero.org/google-docs/?XeCLW4)) showed that taking part in the interventions resulted in the increase of parenting knowledge [[46]](https://www.zotero.org/google-docs/?WS9QPM) or knowledge about early childhood cognitive and language development [[47–49]](https://www.zotero.org/google-docs/?xMP7bO). In most cases ([[48,49]](https://www.zotero.org/google-docs/?waQOk5) and high intensity training described by List and collaborators [[47]](https://www.zotero.org/google-docs/?YqB3CT)) increase in knowledge was also reported to be connected to enriched parent-child interactions.

We also added additional parts to the existing paragraph of the same section (lines 139-141).:

One such intervention that has proven to enhance both parental responsiveness and children’s outcomes, including early language skills, is *Parents As Literacy Supporters (PALS)* [[44]](https://www.zotero.org/google-docs/?i7jqHs).

and (lines 149-155):

PALS was later adapted to various settings. For example, Feil and collaborators [[46]](https://www.zotero.org/google-docs/?X7XxKd) adapted the program to be internet-based and provided it to a diverse group of participants (38.7% of whom were Hispanic mothers). The study included a control group and showed that mothers who participated in PALS demonstrated a significantly greater increase in parenting knowledge compared to those in the control group, who received attention-control training. The experimental condition also improved maternal language-supportive behaviors as well as children's language outcomes.

## Comment 2

**Comment of the Reviewer:**

Recruitment and randomization procedures are well described but the method section would benefit from a clearer organization. First of all, a section devoted to measures would be useful. Here we need more information about the content in the researcher developed measures of parent beliefs with examples of items, as well as its psychometric properties. Did the authors run exploratory factor analysis to confirm that all items load on the same factor or is the questionnaire tapping into different constructs relating to parental beliefs? If more than one, what are scores on different constructs and correlations? What is the internal consistency of the scale? Were any of the co-variates related to this index at pretest?

**Response:**

Thank you for this valuable comment highlighting the need for clearer information in the measures section. We have made revisions to this part of the manuscript (now titled **Variables and Measures**) and added more detailed information about the parental beliefs questionnaire (Bajkowska et al., 2022/unpublished). Specifically, we have included example items as well as details regarding its psychometric properties (e.g., internal consistency). These descriptions are based on a pilot study conducted with a larger initial set of items (N = 88). The number of participants in the pilot study (n = 98) was however far too small for us to be able to examine the factorial structure properly. In a separate study (independent of this described in the manuscript and carried out when the current study was already in progress), we carried out a validation study of the final version of the questionnaire on a larger sample of participants, which will enable us to examine its factorial structure comprehensively. The data from this validation study are currently being analyzed, and we hope to publish a separate article focusing exclusively on all stages of developing and validating this questionnaire. We believe these additions improve clarity and address the reviewer’s concerns.

We also wanted to thank you for pointing out the need for including the information about the correlations between the co-variates and the results that the participant got in the pretest.

We also added information about the correlation between co-variates used in the regression model (education, number of children and engagement) and the score at the parental beliefs questionnaire at pretest in the Results section. We found the preexisting correlation between the score on beliefs questionnaire (at pretest) and participant’s education to further limit the possibility of drawing conclusions on the lack of the association between the education and the gain from taking part in the intervention (as existing association might have been hidden in the fact that score at the pretest was the first, strongest predictor in the model explaining the gain from the intervention). We added the info on that to the *Discussion* section.

**Change in the manuscript:**

We renamed the subsection (of the *Methods* section) *Parental questionnaire and variables measured* to *Variables and Measures* and modified the content. In particular, we included additional information on the *Questionnaire on parental attitudes towards language development and bilingualism* (lines 424-432):

Sample items include: “Speaking to infants in a specific way (e.g. in a higher tone of voice, with exaggerated intonation, using diminutives) helps them learn to speak,” (correct answer: agree; ; 2 points given for selecting “I agree” or “I totally agree”) and “Leaving the TV/radio on in the background is a great way to give 0- to 2-year-olds extra chances to learn new words.” (correct answer: disagree; 2 points given for selecting “I disagree” or “I totally disagree”). The scoring system described yielded a total score ranging from 0 to 62 points. The questionnaire has good internal consistency (Cronbach’s alpha = .83, based on the pilot study conducted on a larger number of items (*n* = 98) [[63,64]](https://www.zotero.org/google-docs/?6bI9B7).

The parts added due to inclusion of the correlation analysis between co-variates used in the regression model (education, number of children and engagement) and the score at the parental beliefs questionnaire at pretest:

In the *Analytic strategy* section (lines 482-487):

To further investigate the relationship between the predictors and the increase in parental beliefs alignment with scientific evidence, we first examined bivariate correlations between each predictor and the initial level of parental beliefs alignment (TP1 score). This allowed us to assess the extent to which the predictors were related to the baseline values. The correlations are considered supplementary to the primary regression analyses and, as such, were not specified in the preregistration.

In the Results section (lines 560-563):

The *number of children in the household* was significantly positively correlated with the pretest parental beliefs alignment score, *r*(46) = .36, *p* < .001. A similar positive association was found for parental education, *r*(46) = .31, *p* = .004. No significant correlation was observed between parental engagement and the TP1 score.

In the Discussion section (lines 672-676):

Another possible explanation for why education did not significantly predict gains from the intervention is that, at the start of the study (TP1, in the pretest), it was already positively correlated with parental beliefs. The pretest score was the strongest predictor of the participants’ gain from taking part in the intervention, which may have obscured the effect of education level on intervention gains.

## Comment 3

**Comment of the Reviewer:**

Secondly, more information about the engagement coding would be useful for interpreting these results. I assume that the engagement measure is tapping into (an aspects of) parents’ fidelity of the intervention, but what type of behaviors were coded and how.

However, it would be useful with information about how many parents completed all workshops. If there is any variation here, this could be an interesting predictor too.

**Response:**

Thank you for pointing out that the engagement measure was not clearly described. In the *Variables and Measures* section we added information about all measures used as predictors in the regression model and how they were collected. We focused on the details of the coding procedure for the engagement measure. As you will see, the measure reflected how many sessions the participant attended.

We also added the information on how many participants completed what number of sessions during the intervention.

**Change in the manuscript:**

Information about the engagement coding added to *Variables and Measures* subsection of the *Method* section (lines 440-453):

The first two of those variables (education and number of children) were collected in the demographic questionnaire. The third one, parental beliefs at the study entry, was assessed using the same questionnaire as the dependent variable [[60]](https://www.zotero.org/google-docs/?tuGnzl). The last variable (participant’s engagement in the intervention) was measured by the trained observer as the number of times that the participant spontaneously spoke during the workshop: e.g. shared the experience after the exercise or asked a clarifying question. Whenever the participant spoke, the observer noted this fact in the observation sheet. The participant’s words were not noted, just the simple fact of speaking. Participants were informed about the presence of the observer and her task before the workshops began. The final value of the engagement variable for each participant was calculated as the sum of the number of verbal contributions across all sessions combined. In this way, the engagement variable reflected whether the participant attended each session (as missing a session precluded obtaining any activity points for that meeting) as well as whether their participation went beyond passive listening.

Information on how many participants completed what number of sessions during the intervention added to the *The group recruited for the study* subsection in *Participants* section (lines 315-327):

Out of the 95 participants who attended at least one of the three intervention sessions in person, 84 completed both the pretest and posttest measures and were therefore included in the current analyses. The final randomised sample consisted of N = 76 randomised participants; N = 40 in the experimental group and N = 36 in the control group followed by *N =* 8 additional non-randomised participants included only in the additional, exploratory analyses (thus *N =* 84 in total).

Among these 84 participants, 55 attended all three sessions synchronously online: 31 in the experimental group (including 24 from the randomised trial) and 24 in the control group). Nineteen participants attended two out of three sessions: 11 in the experimental group (all from the randomised trial) and 8 in the control group. Ten participants attended only one session in person: 6 in the experimental group (including 5 from the randomised trial) and 4 in the control group. All participants were given the opportunity to update themselves on the material independently using handouts provided in pdf.

## Comment 4

**Comment of the Reviewer:**

There is some mentioning of the analytic strategy in the section Parent questionnaire and variables measured and in the Results section but I think this information is better placed in an analytic strategy section. In that [analytic strategy] section, more information about choice of model would be good. Both the mixed ANOVA and the linear regression used in the moderation analysis should be described in the Analytic section.

**Response:**

We added the section Analytic Strategy. We moved there the information that was previously placed in the Parent questionnaire and variables measured subsection and some information from the Results section. We also added some more details to make our analytic strategy clear.

**Change in the manuscript:**

We added section Analytic Strategy (lines 455-499):

## **Analytic strategy**

The article presents three analyses. Two of them – the mixed ANOVA testing the effect of the intervention and the regression analysis identifying predictors of increased alignment between participants’ beliefs and scientific evidence – were preregistered (https://osf.io/kfgba/?view_only=41fe68b36b7a4f33ab5ddb19c50adecc). The third analysis – a mixed ANOVA examining whether the effects of the intervention were sustained over time – was not included in the preregistration, as it had not yet been planned at that stage.

As planned in the preregistration, we conducted a mixed ANOVA to test **the effectiveness of the intervention.** The score on the attitudes questionnaire (Bajkowska et al., 2022/unpublished) was a dependent variable and group and time point as independent variables. Group assignment (experimental or control) was treated as a between-subject variable and time point (pretest – TP1 vs. posttest – TP2) was a within-subject variable. As the groups did not differ on the levels of control variables (measured by the two sample t-tests: one for the education level: *t(71)* = 0.05, ns; and one for the number of children in the household: *t(74)* = 0.78, ns) there was no need to include any co-variates to the model.

In the **exploratory analysis,** linear regression was conducted to verify what were t**he best predictors of the increase in the alignment of participants’ beliefs with scientific evidence**. This analysis was also included in the preregistration. The dependent variable was the increase in the score on the attitudes questionnaire (Bajkowska et al., 2022/unpublished) between pretest (TP1) and posttest (TP2). This increase was calculated as the difference of scores between TP2 and TP1 scores. The independent variables were: 1) the level of scientifically based beliefs at study entry (TP1 score); 2) the number of children in the household; 3) the level of engagement in the intervention; and 4) the level of each participant’s education. For the purpose of this analysis, the level of the participant’s education was turned into a number and treated as a numeric variable (primary school –1, middle school to vocational education – 2, secondary school – 3, incomplete higher education – 4, tertiary education – BSc; BA; Eng.; MSc; MA – 5, tertiary education **–** PhD – 6).

To further investigate the relationship between the predictors and the increase in parental beliefs alignment with scientific evidence, we first examined bivariate correlations between each predictor and the initial level of parental beliefs alignment (TP1 score). This allowed us to assess the extent to which the predictors were related to the baseline values. The correlations are considered supplementary to the primary regression analyses and, as such, were not specified in the preregistration.

To test **if the effect of the intervention persisted over time**, we conducted a mixed ANOVA with the score on the attitudes questionnaire (Bajkowska et al., 2022/unpublished) as a dependent variable and group and time point as independent variables. This analysis was not included in the preregistration. Group assignment (experimental or control) was treated as a between-subject variable and time point (pretest – TP1 vs. posttest – TP2 vs. retention test – TP3) was a within-subject variable. As the groups did not differ on the levels of control variables (measured by the two sample t-tests: one for the education level: *t(29)* = –1.22, ns; and one for the number of children in the household: *t(31)* = 1.26, ns) there was no need to include any co-variates to the model.

All the data were analysed in R Statistical Software (v4.4.0; R Core Team, 2024). The data and the script are available at OSF (https://osf.io/kfgba/?view_only=41fe68b36b7a4f33ab5ddb19c50adecc).

## Comment 5

**Comment of the Reviewer:**

It is not clear to me why the non-randomized language workshop group is included in the moderation analysis (not least as the effect measures are different).

**Response:**

Thank you for pointing out that the description of the non-randomised group was not sufficiently clear. This group underwent exactly the same intervention and completed the same assessments as the randomised experimental group. For this reason, we decided to include these participants in the analysis exploring predictors of the degree of benefit gained from the intervention, in order to increase statistical power.

The only difference between the groups was related to the recruitment process: participants in the non-randomised group were informed from the outset that they were enrolling in a study that involved a workshop specifically focused on supporting early bilingual development. Consequently, it was not possible to randomly assign them to a control condition. In contrast, the randomised participants were informed that they would take part in a workshop about supporting early child development in general, without specifying the detailed topic of the intervention, which enabled random allocation to the experimental or control group. We acknowledge that this distinction was not clearly described in the original manuscript, and we have now added clarifying information to address this point.

**Change in the manuscript:**

We included more information about the non-randomised group in the subsection *The group recruited for the study* of *Participants* section (lines 288-304):

Despite extensive efforts, only 8 participants were recruited, and they were assigned to an additional experimental group (language workshop), which made it unfeasible to proceed with the planned comparison between face-to-face and online formats. As a result, we decided to offer these participants the same online workshop as those in the randomized trial. These participants were not included in the main analyses testing the effectiveness of the intervention, as they were not randomly assigned to groups. They enrolled in the study with the understanding from the outset that they would participate in the workshop related to child language (as opposed to the randomised participants who were informed that they would take part in a workshop about supporting early child development in general). However, ultimately, they received the exact same experimental (language) intervention – delivered in the same format – as the participants in the randomised trial, and they completed the same set of questionnaires. For this reason, we decided to include them in an additional exploratory analysis aimed at investigating factors associated with the degree of benefit gained from participation in the experimental intervention to boost the statistical power of this analysis. This subgroup was not originally planned and was therefore not included in the preregistration. The subgroup is referred to in the article as a non-randomised group.

## Comment 6

**Comment of the Reviewer:**

Also, exact description of attrition is lacking (and n is not reported in tables). Judge from plots there is a high attrition rate between T2 and T3 but how was the missingness handled in the analyses.

**Response:**

The information was previously stated in the Results section (currently lines 577-580):

The data from participants who were assigned to groups randomly and filled in the questionnaire at all three time points: pretest, posttest and retention test (when the children were 9 months-old) were included in this analysis (N = 42, n = 17 in the experimental group, and, n = 25 in the control group).

We acknowledge that the description of the attrition was lacking in the *Participants* section and the number of participants whose data were included in this analysis were lacking in Table 4. We corrected this omission.

**Change in the manuscript:**

We added information about the sample used in the retention analysis in the subsection *The group recruited for the study* of *Participants* section (lines 335-341):

In the analyses regarding the long-term effects of the intervention (retention of the changes in parental beliefs), we included only data from participants from the randomised groups who completed the beliefs questionnaire at all three time points: before the intervention (TP1), after the intervention (TP2), and when the children were 9 months-old (TP3). The drop-out rate at the last stage of the study was substantial, so the sample in this analysis was much smaller (*n* = 42; *n* = 17 in the experimental group, and *n* = 25 in the control group) than the one in the analysis corresponding to the first research question.

and in the Table 4 (Descriptive statistics for participants’ questionnaire score at pretest (TP1), posttest (TP2) and second posttest (when the child was 9 months old, TP3) in the experimental and in the control group):

| Group | TP1 | | TP2 | | TP3 | |
| --- | --- | --- | --- | --- | --- | --- |
|  | *M* | *SD* | *M* | *SD* | *M* | *SD* |
| Experimental – language workshop  *n* = 17 | 45.0 | 9.83 | 51.9 | 6.99 | 52.6 | 6.50 |
| Control – sleep workshop  *n* = 25 | 41.4 | 9.43 | 44.3 | 8.02 | 45.8 | 7.62 |

We also added the information about this in the *Limitations* section (lines 718-719):

Another limitation is the relatively small size of the study sample (especially at TP3), due to the recruitment difficulties and drop-out rate at each stage of the study.

## Comment 7

**Comment of the Reviewer:**

I also lack information about if and which co-variates is used as control variables in the main model and the moderation model.

**Response:**

There were no co-variates used in the models. There were variables that we found potentially influencing the results for the main model: parent’s education and the number of children in the household. These variables were used in the pseudo-randomisation. The participants were assigned to groups in the way that would minimize the imbalance in the levels of these variables between groups (as described in *The group recruited for the study* subsection in the *Participants* section). The third variable was the child’s expected sex, which could be an important factor at later stages of the study, including the observation of children’s language outcomes. As the process of pseudorandomisation was successful (group means for the controlled variables did not differ and were very close to each other), we did not find the need for including any co-variates to the main model.

In the analysis searching for the predictors of the gain from taking part in the intervention, all the variables that we expected may influence the dependent variable were treated as independent variables, so we also did not see the need for including the co-variates in this analysis.

We included the information about the fact that the groups did not differ on the control variables in the *Analytic strategy* section.

**Change in the manuscript:**

We included information regarding no need for co-variates in the main model in the *Analytic strategy* section (lines 466-469):

As the groups did not differ on the levels of control variables (measured by the two sample t-tests: one for the education level: *t(71)* = 0.05, ns; and one for the number of children in the household: *t(74)* = 0.78, ns) there was no need to include any co-variates to the model.
Same was done for the analysis of the retention of gain from taking part in the intervention (lines 493-496):

As the groups did not differ on the levels of control variables (measured by the two sample t-tests: one for the education level: *t(29)* = –1.22, ns; and one for the number of children in the household: *t(31)* = 1.26, ns) there was no need to include any co-variates to the model.

## Comment 8

**Comment of the Reviewer:**

I wonder how the fact that all participants come from one language background affect the generalizability of the results. It would be good to hear the authors reflections on this.

**Response:**

Thank you for raising this important point. We agree that the fact that all participants came from a single language background (Polish-speaking families living in Norway) has implications for the generalizability of the findings. We now address this issue more explicitly in the *Limitations* section of the manuscript. We also decided to include the information about the language background to the title of the manuscript.

**Change in the manuscript:**

We added the reflections on that matter to the *Limitations* section (lines 723-744):

Finally, the sample used in this study was relatively homogeneous, which may limit the generalizability of our findings. On the one hand, focusing on Polish-speaking participants living in Norway offers a clear advantage, as it enables a detailed examination and comparison of children’s language development in both languages at later stages of the study. On the other hand, this raises questions about the extent to which our results are applicable to other populations – including Polish immigrants in different countries, and even more so to parents raising bilingual children in more diverse contexts – for example, in countries with multiple distinct official languages, such as Luxembourg, or among refugee families whose migration is driven by war rather than economic reasons.

The fact that the sample was limited to Polish speaking parents in Norway might also influence the other characteristics of the sample, such as the fact that it consisted mainly of well-educated Polish women living in Norway, with no participants reporting only primary education. While this group may be representative of young Polish female migrants in Norway, 47% of whom hold a university degree and only 14% have a high school education or lower [[69]](https://www.zotero.org/google-docs/?LDKxI8), this demographic homogeneity may limit the applicability of our findings to more diverse populations, including less-educated parents or those in different socio-cultural contexts.

We translated the intervention content and materials to English and Norwegian and the translations along with the Polish original are publicly available on the OSF (<https://osf.io/46fvq/?view_only=8a4ddeb5840d4a09894ca0df2b7dcebe>). We hope that this can lead to other studies, examining the efficiency of the intervention in more diverse settings.
