# Supplementary material for: Better start to bilingual development: Bridging parental beliefs and science through early intervention for Polish families living in Norway
Source: PLoS One. 2026 Apr 9;21(4):e0346160. doi: 10.1371/journal.pone.0346160 (PMC13065072; doi:10.1371/journal.pone.0346160)
Supplement: Attachment — Submitted filename: Polish in Norway.docx [file pone.0346160.s002.docx]

How can both an IV and a DV use the same measure? It could be pre- post for that third variable but at entry isn’t it the same?

Table 2 descriptive statistics on what variable?

I think you mean a Bonferroni post-hoc (which controls for increasing alpha) and not a Bonferroni correction

686 effect increased with time but

There were no significant differences 605 between the posttest and the retention test. For the control group, no significant differences 606 were observed between any time points

688 effect was seen in the control group but

For the control group, no significant differences 606 were observed between any time points

These two in red from the discussion do not seem to agree with your results

Then

As the effect is found in both the experimental and the 697 control group, general reasons, like being exposed to scientifically based knowledge after 698 giving birth or becoming interested in scientifically based knowledge after attending 699 workshops, cannot be ruled out as explanations.

I don’t see the effect in the control group

Data—please provide the ANOVA tables that match the two analyses that you completed

The regression needs to be more detailed in how you conducted entering the predictor variables. The best one and would add to your paper is hierarchical where you add the ones that have the highest prediction first and then one by one after that. The benefit of this is that you can get R square change scores. In that way you can tell how much variance is explained by the intervention itself and then how much by having other children etc. It provides much more information.
