# Supplementary material for: Better start to bilingual development: Bridging parental beliefs and science through early intervention for Polish families living in Norway
Source: PLoS One. 2026 Apr 9;21(4):e0346160. doi: 10.1371/journal.pone.0346160 (PMC13065072; doi:10.1371/journal.pone.0346160)
Supplement: Attachment — Submitted filename: Polish bilinguals.docx [file pone.0346160.s004.docx]

The paper is much improved but includes sections and statements that do not provide new knowledge

The Introduction section of this manuscript is one of those sections. I suggest removing it.

The statement before is on lines 84-86. It basically repeats the sentence above it. Delete

Parents' childcare 85 practices are influenced by their knowledge about language development and their attitudes 86 towards various communication and caregiving behaviours [17]

Line 128---add ‘and’ before *Comment*

**Evidence-based recommendations for parents and parental interventions**—this heading is not needed---the section is extremely short and can be one paragraph and integrated into the section before this heading

This paragraph is basically redundant to other sections:

Most of the research on parental interventions available in the literature is addressed 138 to monolingual populations. Some studies describe language-related interventions aimed at 139 bilingual families of low SES [e.g. Latino communities in the US, 38], or where multilingual 140 families constituted a significant proportion of participants [e.g. 39–42]. However, the studies 141 on bilingual families focused solely on the majority language of the children. Hence, 142 language interventions aimed at preventing language difficulties have yet to focus on families 143 planning to raise children in bilingual environments [4

Some parts do not have citations (i.e., first sentence)

Again, the heading is not needed nor is the above paragraph (138 to 143)

Then the next paragraph makes your case but you stated that a few studies and you do not cite them. Please add citations to that statement

163 to 170 discusses a topic you hsd earlier---please move it to that area

This sentence is also redundant:

This is consistent with the studies showing that parental 177 knowledge about language and cognitive development at the time of their child’s birth 178 influences caregiving behaviours several months later [17]

This one is redundant of the sentence before it:

Dialogic reading has been implemented as 194 a standalone approach in numerous parent-oriented language interventions.—delete

197 to 201 is again redundant –please delete

Delete redundant:

Our intervention was developed based on the research on communication 210 and caregiving behaviours supporting language development

Not a paragraph:

We hypothesised that the alignment of parental beliefs with scientific evidence on 232 language development would increase significantly in the group that received the language 233 intervention compared to the control group.

If it adds new information, integrate it into the paragraph below

Line 267 birthing (how it is discussed in English)

On 338 you said of the 95—84 attended at least one session in person

Then in the next paragraph you state—of the 84—55 attended three synchronously

Please make this into a table as it is difficult to follow as written

Line 460 (Cronbach’s alpha = .83) please add the )

Regressions do not have IV and DVs. They had predictors and outcomes—please rephrase

you have some squares in the stats (line 547) but when I copy it is comes out as 𝛽-squared---please check

674---given that only the first model added do not include the other models as they are not contributing to the effect

No data in the discussion---the discussion is not a repeat of the results so interpret the importance of the findings in relationship to the lit review and any other possible new discoveries

You have multicollinearity in your data—in 868

From AI

a statistical phenomenon in regression analysis where two or more independent variables are highly correlated, meaning one can predict another, creating redundant information that makes it difficult to determine each variable's individual effect on the [outcome variable](https://www.google.com/search?q=dependent+variable&oq=definition+of+multicolnear&gs_lcrp=EgZjaHJvbWUqCQgBEAAYDRiABDIGCAAQRRg5MgkIARAAGA0YgAQyCAgCEAAYDRgeMggIAxAAGA0YHjIKCAQQABgIGA0YHjIHCAUQABjvBdIBCTE5MzcxajBqN6gCALACAA&sourceid=chrome&ie=UTF-8&ved=2ahUKEwi_-u-189GRAxUYvokEHSE6JUMQgK4QegYIAQgAEAY)

it is like parental income and free lunch in the US. To get free lunch – parents have to have low income—so they are the same variable
